# Polydeoxyribonucleotides Pre-Clinical Findings in Bone Healing: A Scoping Review

**DOI:** 10.3390/dj11120280

**Published:** 2023-12-04

**Authors:** Mattia Manfredini, Pier Paolo Poli, Mario Beretta, Matteo Pellegrini, Federica Eugenia Salina, Carlo Maiorana

**Affiliations:** 1 Department of Biomedical, Surgical and Dental Sciences, University of Milan, 20122 Milan, Italy; mattia.manfredini@unimi.it (M.M.); mario.beretta@unimi.it (M.B.); matteo.pellegrini@unimi.it (M.P.); federicaeugenia.salina@studenti.unimi.it (F.E.S.); carlo.maiorana@unimi.it (C.M.); 2 Implant Center for Edentulism and Jawbone Atrophies, Maxillofacial Surgery and Dental Unit, Fondazione IRCCS Ca’ Granda Ospedale Maggiore Policlinico, 20122 Milan, Italy

**Keywords:** biopolymers, bone regeneration, dental implants, dentistry, polydeoxyribonucleotide, polynucleotides

## Abstract

Aim: Polydeoxyribonucleotide (PDRN) is a chain-like polymer derived from DNA. Recent in vitro and animal studies have showcased the beneficial impacts of PDRN on the process of bone mending, whether used on its own or in conjunction with other substances that aid in regeneration. This scoping review aims to synthesize the current understanding of how PDRNs influence bone healing. Materials and Methods: The studies included in the screening procedure were randomized controlled clinical trials (RCTs), both retrospective and prospective case–control studies, as well as in vitro and in vivo investigations. Articles were sourced from PubMed (MEDLINE), Scopus, EMBASE, Web of Science, and Google Scholar electronic databases using the following MeSH terms: (polydeoxyribonucleotide) and (bone) and (regeneration). Results: Initially, 228 articles were identified. Following the review process, a total of eight studies were ultimately examined. Among these, two were confined to laboratory studies, five were conducted on living organisms, and one encompassed both evaluations on living organisms and in vitro assessments. A descriptive qualitative approach was employed to present the data extracted from the studies that were included. Conclusions: PDRN has the potential to enhance the process of bone healing and the quantity of newly generated bone when combined with grafting materials. Future clinical studies are warranted to ascertain the appropriate clinical application of PDRN based on the dosage under consideration.

## 1. Introduction

Polydeoxyribonucleotide or polyideribotide (PDRN) is a linear DNA-derived polymer with healing activity used in the treatment of skin and connective tissue lesions associated with dystrophic and dystrophic-ulcerative diseases. It consists of a mixture of purines, pyrimidines, deoxyribonucleotides, and deoxyribonucleosides with different lengths (50–2000 base pairs), and a molecular weight between 50 and 1500 kDa [1].

PDRN is typically sourced from the gonads of salmon trout (Oncorhynchus mykiss) due to their provision of high-grade DNA without the presence of pharmacologically active proteins and peptides, thereby mitigating potential associated adverse effects. [2]. After extraction, a purification and sterilization process is performed, achieving >95% purity [3].

PDRN is available in the pharmaceutical market in the form of vials for parenteral administration, as well as in the form of eye drops and ointment. After administration, it can be detected freely in the plasma, exhibiting a bioavailability within the range of 80–90% [4]. Its concentration peaks around one hour post-administration, and it possesses a half-life of approximately 3.5 h. Importantly, PDRN does not undergo hepatic metabolization; instead, it is broken down by non-specific plasma or membrane nucleases. Ultimately, the substance is excreted primarily through urine, with a smaller proportion being eliminated via feces [4].

Enzymatic breakdown of PDRN produces biologically active byproducts, including oligo- and mononucleotides, purines, and pyrimidines. These substances interact with purinergic adenosine A2A receptors (ADORA2A), prompting wound healing by facilitating cell migration and proliferation, ensuring proper deposition of the extracellular matrix, stimulating angiogenesis, and reducing inflammation [5]. The administration of dimethyl-1-propargyl xanthine (DMPX), a selective ADORA2A antagonist, counteracts these effects, enhancing the safety profile of PDRN compared to other ADORA2A agonists [6]. The combination of high therapeutic effectiveness, a low likelihood of provoking an immune response, and a lack of adverse effects, regardless of the method of administration, allows for the utilization of PDRN in patients with compromised health, such as those with diabetes [6].

Furthermore, PDRN facilitates DNA synthesis and repair, reinvigorating cellular proliferation and growth in damaged or oxygen-deprived tissues through the “salvage pathway”. This metabolic route allows PDRN to supply cells, which are unable to independently generate new DNA, with nucleotides sourced from its breakdown [3].

Currently, PDRNs find application in the treatment of conditions affecting bone, cartilage, and tendons [7]. Recent studies conducted in vitro and on animals have demonstrated their beneficial effects on bone healing, particularly in the presence of bone defects, either when used alone or in combination with other regenerative materials [8,9].

The goal is to enhance bone healing with the aim of reducing biological timeframes for regeneration, thus providing prosthetic solutions to patients more rapidly while minimizing the invasiveness of the procedure and reducing the need for autologous bone grafts.

Given the critical need to enhance the process of bone healing in oral and maxillofacial surgery, it is imperative to explore which compound may provide superior benefits in terms of the speed of new bone formation, the attachment of bone grafts, and overall post-surgical recovery, this scoping review aims to examine whether the use of PDRN can improve bone healing in oral surgery with analysis of in vitro, animal, and clinical studies.

## 2. Materials and Methods

### 2.1. Protocol

This scoping review adhered to the guidelines outlined in the Preferred Reporting Items for Systematic Reviews and Meta-Analyses extension for Scoping Reviews (PRISMA-ScR) to comprehensively synthesize existing evidence and pinpoint key concepts regarding the application of PDRNs in bone regeneration (Table S1 Supplementary Materials) [10].

The present protocol has been registered within the Open Science Framework platform (Registration DOI-10.17605/OSF.IO/MCBZD).

An adapted version of the PICO (Population, Intervention, Comparison, and Outcome) model was employed to formulate a focused question structured around a PEO (Population, Exposure, and Outcome) framework. This approach was utilized to assess the relationship between a specific exposure and its resulting outcomes. It was originally designed for conducting qualitative systematic reviews of healthcare interventions, encompassing procedures in oral surgery as well [11,12].

The main question was, “Does PDRN improve the ability to regenerate bone in the oral environment?”. To answer this question, studies reporting on bone regeneration following PDRNs application were analyzed with the aim to understand the possible impact PDRNs have on bone healing.

### 2.2. Eligibility Criteria

#### 2.2.1. Inclusion Criteria

All sources of evidence had to satisfy specific inclusion criteria to be included. These criteria encompassed articles written exclusively in English and did not impose any restrictions based on publication date. The screening process encompassed randomized controlled trials (RCTs), as well as retrospective and prospective case–control studies. Both in vivo and in vitro studies were incorporated in this review.

The primary focus of the investigation in these studies was to assess the bone regenerative effects of PDRN, with a particular emphasis on experimental research.

#### 2.2.2. Exclusion Criteria

Any studies that did not meet the specified inclusion criteria were excluded from the review. This included articles written in languages other than English, case reports, literature reviews, and studies that primarily focused on the healing of soft tissues following the application of PDRN.

#### 2.2.3. Search Strategies and Information Source

To perform this review, the PEO model (Population, Exposure, and Outcome) was used through a literature search of the electronic databases PubMed, Scopus, EMBASE, Web of Science, and Google Scholar databases.

The PEO model [13] is based on the two elements population (in this case, the review was not limited to a specific population) and exposure (evidence from in vivo and in vitro clinical trials related to the potential PDRN employment in bone regeneration).

Specific Medical Subject Headings (MeSH) were employed to search through all electronic databases to locate pertinent studies in alignment with the exact parameters of the PEO query. Articles were chosen from electronic databases based on specific MeSH terms, including polydeoxyribonucleotide, bone, and regeneration. Consequently, a consistent search strategy was applied to screen publications across all electronic databases, structured as (“polydeoxyribonucleotide” (MeSH)) AND (“bone” (MeSH)) AND (“regeneration” (MeSH)).

Further examination of the reference lists of all relevant articles was conducted, but no additional pertinent studies were discovered. It is important to note that no filters were applied to each search string during the electronic research process. The years from 1968 to March 2023 were considered in all databases. The last search was performed on 20 August 2023.

#### 2.2.4. Selection of Sources of Evidence

Two independent reviewers (F.E.S. and M.P.) carried out the initial screening of titles and abstracts for all included articles. Any duplicate entries in the databases were identified and subsequently eliminated using the EndNote Web reference manager software (version 20) by Clarivate Analytics, based in Philadelphia, PA, USA. Full-text articles were then individually assessed, with the results duly recorded, and any similar studies meeting the inclusion criteria were identified. The two researchers compared their selections, and in case of any discrepancies, they were brought to the attention of the other four researchers (M.M., M.B., P.P.P. and C.M.) for resolution.

#### 2.2.5. Methodological and Reporting Quality Assessment

Since this scoping review aims to map the scientific literature on the role of PDRN in bone healing by synthesizing all studies published to date, i.e., in vitro and in vivo studies, in accordance with the PRISMA-ScR guidelines, the quality assessment of the included studies was not performed.

#### 2.2.6. Analysis of Included Studies

Following the review of the publications, a spreadsheet was generated and subsequently updated in a sequential manner. Two separate tables were created, one for in vivo studies and one for in vitro studies. For the in vitro studies, the collected data were organized into tables, which provided a structured presentation of the information: the name of the first author of the article and the year of publication, the type of PDRN used, the experimental groups investigated, the follow-up period, analyses performed on the samples, and the results of this analysis. Analyses performed on the samples means the explanation of how some variables, such as stimulation of osteoblasts by PDRN, were evaluated and analyzed in the in vitro studies.

As for the in vivo studies, the gathered data were organized into tables, allowing for a systematic presentation of the information: the name of the first author of the article and the year of publication, the animal model, the type of study, the type of PDRN used, the experimental groups investigated, the follow-up period, complications that occurred during the healing period, analysis of newly formed bone volume, and qualitative histological analysis.

## 3. Results

Initially using MeSH terms, 9 articles were identified in PubMed, 19 in Embase, 17 in SCOPUS, 18 in the Web of Science, and 165 in Google Scholar.

After removing duplicates, 177 articles were left for initial screening. Upon reviewing titles and abstracts, 166 publications were deemed ineligible and subsequently excluded. The full texts of the remaining 11 articles were carefully examined. Out of these, three studies had to be excluded after a thorough review of the full texts, as they did not meet the inclusion criteria. Specifically, one article [8] was excluded because sodium-DNA was used instead of PDRN, and the other two articles because they were concerned with the effectiveness of PDRN in the management of osteonecrosis [2,14]. Finally, eight studies [1,15,16,17,18,19,20,21] were included after the review process.

**Figure 1:**
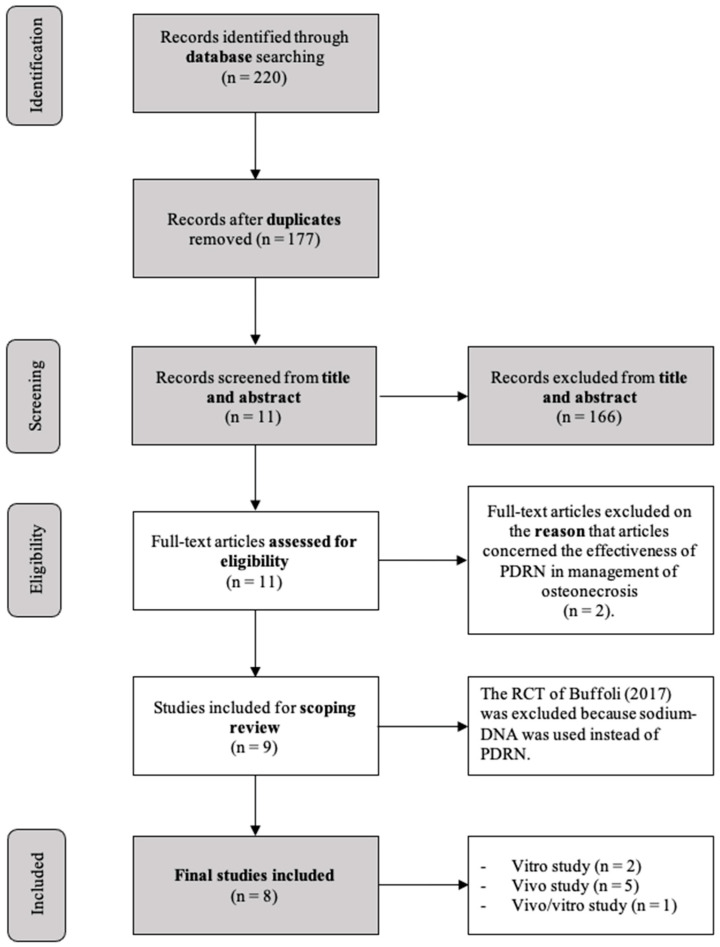
Flowchart of the review process [8].

The selected studies included two in vitro studies [15,18], five in vivo studies [1,16,19,20,21], and finally, one study that reported both in vivo and in vitro evaluations [18]. The flowchart of the review process is shown in Figure 1. All included studies were RCTs except one animal case series.

### Results of Individual Sources of Evidence

A meta-analysis of the selected articles could not be carried out due to the differences in the type of studies included (in vivo and in vitro) and within the same type of studies, the difference between the treatments and the commercial formulations of PDRN. A qualitative descriptive approach was employed to present and summarize the collected data. Data collection and study outcomes are contained in Table 1 for in vitro studies and Table 2 for in vivo studies.

## 4. Discussion

The long-term success rate of endosseous implants is ensured by adequate bone volume at the recipient site. In the presence of bone defects caused by atrophy, dental trauma, extractions, or periodontal disease, regenerative surgical procedures are required before implant placement [22]. Among the different surgical procedures described to augment the bony envelope for implant placement purposes, guided bone regeneration (GBR) showed promising results in the long term. GBR involves using a barrier membrane placed over a bone defect or extraction site to promote the selective growth of osteogenic cells and prevent defect colonization by soft tissue [22].

The barrier effect in combination with blood clot alone requires a significant amount of time to regenerate even limited amounts of bone. Thus, autologous bone grafts eventually combined with biomaterials were introduced as filling materials to improve efficacy and reduce the healing time of the regenerative process [23]. Autogenous bone is considered the gold-standard grafting material due to its excellent osteogenic and osteoinductive properties associated with the highest biocompatibility. Nevertheless, main drawbacks are the limited intraoral availability and the need for a second surgical site for harvesting [24]. To overcome these limitations, allogeneic, xenogeneic, and synthetic bone substitutes and graft materials based on extracted teeth, which exhibit osteoconductive and osteoinductive properties, have been developed and clinically used [24,25,26]. Such materials are osteoconductive but not osteoinductive; therefore, the association with autologous grafts is suggested [27].

To further improve the regenerative outcome, the search for molecules that can stimulate osteoblastic proliferation remains a topic of interest in oral surgery. Such biomolecules can be used in bone defects mixed with biomaterials to stimulate faster osteoblastic proliferation, and, consequently, reduce healing time with the formation of new bone [28]. To this aim, PDRNs are being assessed in vitro and in animal models to promote bone healing and improve hard tissue regeneration.

In one of the initial in vitro studies, the researchers investigated the potential of PDRNs to stimulate growth and enhance the activity of cultured human osteoblasts that were isolated from the jawbone following surgery. This was achieved by increasing the synthesis of alkaline phosphatase [15]. The study demonstrated that osteoblasts treated with PDRN (at a concentration of 100 μg/mL) exhibited an optimal and significant growth rate when exposed to a 10% concentration of fetal bovine serum (FBS), with noticeable effects as early as 48 h and peaking at 6 days. This resulted in a 21% increase in cell count. In contrast, cultures lacking FBS showed no discernible impact from PDRN. The authors explained this phenomenon with the presence of enzymes that, through a depolymerization process, generated purine nucleotides and free nucleosides capable of binding to purinergic receptors [15].

Furthermore, the results obtained from treatment with DMPX and Suramine (inhibitors of purinergic A2 and P2 receptors, respectively) indicated the involvement of A2 receptors in the stimulation of osteoblastic growth by oligonucleotides produced through cellular catabolic degradation. The findings also suggested the likely absence of a role for purinergic P2 receptors. Additionally, they proposed that the adenosine A2 receptor may not be the exclusive mechanism of action of PDRNs [15].

Regarding the assessment of alkaline phosphatase activity, the authors observed an increase in activity in cells treated with PDRN. However, after 10 days, the activity levels were comparable between treated and untreated cells. Since alkaline phosphatase synthesis takes place only in the last G phase before the M phase of the cell cycle, a PDRN-induced rise in cell proliferation led to a reduction in the G phase. These findings suggest that PDRNs may have stimulating effects on osteoblasts and play an important role in the repair of bone defects [15].

For almost two decades, no other in vitro studies evaluated the effects of PDRNs on bone metabolism. In 2021, Kim Da-Seul et al. published two studies using PDRN and biological scaffolds to investigate osteoclastogenesis, osteoconductivity, and the proangiogenic role of PDRN [17,18]. The first study adopted an in vitro approach, while the latter consisted of both in vitro and in vivo models. In both studies, a porous scaffold of Poly(lactic-co-glycolic) acid (PLGA) (P) was associated with a magnesium hydroxide modified with a ricinoleic acid (mMH) (M), and, finally, bovine-derived decellularized bone extracellular matrix (bECM) (E) to create a PME scaffold [17,18]. In the test group of the first study, bioactive polydeoxyribonucleotide (PDRN, P) was incorporated into the PME, creating a PMEP scaffold [17]. In the test group of the second study, the authors designed a PME hybrid scaffold with nano complex (NC), consisting of positively charged bone morphogenetic protein-2 (BMP-2) and negatively charged PDRN [18]. In both studies, mMH showed the exceptional capability of neutralizing the acidic microenvironment created by PLGA degradation. At the same time, bECM, composed mainly of calcium and phosphate, improved the scaffold’s biocompatibility and osteoconductivity [17,18]. In the first in vitro study, the PMEP scaffold demonstrated better hydrophilicity and biocompatibility than the test groups (PME and PLGA) [17]. Furthermore, the PMEP scaffold statistically significantly suppressed the expression of inflammatory genes IL-6 and IL-1*β* and promoted the highest angiogenesis-related gene (VEGF and MMP2) expression compared with the control [17]. Additionally, the PME scaffold induced osteogenic differentiation, but osteogenesis was only enhanced by adding PDRN (PMEP) [17]. Lastly, the bioactive molecules secreted by PME and PMEP scaffolds showed a significant reduction in differentiation into osteoclasts by 31.7% and 74.4%, respectively, compared to the control group (PLGA) [17].

The results obtained in the former analysis were confirmed in the second study [18]. The groups treated with PDRN and NC exhibited enhanced angiogenesis compared to both the control and BMP2-treated groups. Cell proliferation was robust on all scaffolds, with PME/NC showing particularly promising results. Notably, NC demonstrated remarkable osteogenic potential, which was attributed to a synergistic effect of BMP2 and PDRN. Additionally, in groups containing both PDRN and NC, the differentiation of osteoclasts and the expressions of IL-1*β* and IL-6 were statistically suppressed when compared to the control, BMP2, and PDRN groups [18].

In the subsequent in vivo phase, bone defects were created on both sides of rat calvaria using micro drills and trephine burs. In the control group, the defect was left untreated, while in the other two groups, either PME/NC or PME or PLGA were, respectively, placed in the defect. Evaluation of bone regeneration was conducted using micro-CT. The PME/NC-treated group displayed a noticeable increase in bone formation within the defect area, surpassing the other groups in terms of efficacy [18]. The bone volume density, the number of newly formed vessels, and the expression of angiogenetic and osteogenetic genes in the PME/NC-treated defects were significantly higher than those in the other groups [18].

Previous in vivo studies have provided substantial support for the role of PDRN in bone regeneration. An initial animal study investigated the impact of PDRN on the regeneration of cortical bone after creating round defects in 32 rats. The study assessed the performance of three different compounds: PDRN gel, high-temperature protected bone (HDB), and a combination of HDB and PDRN paste [16]. PDRN gel exhibited stimulation of cells and tissues, but its application in gel form during surgery was constrained by challenges in maintaining it at the implantation site. Granular HDB also demonstrated effectiveness; although, the absence of a bonding agent led to some granules leaking out, resulting in ectopic bone formation. Ultimately, the paste composed of HDB and PDRN emerged as a manageable, biocompatible, osteoconductive, and osteostimulating compound that proved applicable for mending bone defects [16]. Histologically, at 12 weeks, no HDB granules were detected outside the surgical defect, and new bone formation was faster than under the other experimental conditions [16].

Subsequently, the effects of PDRNs on hard tissue were examined in beagle dogs. In particular, the bone healing process was evaluated following grafting of xenogeneic bone and anorganic bovine bone in combination or not with PDRNs in bone defects created following immediate post-extractive implant placement [21]. Histological and micro-CT assessments performed at 4 and 8 weeks, respectively, showed that regenerated bone volume in the groups treated with xenogeneic bone and PDRN was greater than that observed in groups treated with xenogeneic bone alone [21]. The authors’ conclusion highlighted that the combination of grafting materials with PDRN has the potential to enhance the bone healing process and lead to an increase in the quantity of newly formed bone over time [21].

Thereafter, in a different animal model, PDRN and human demineralized dentin matrix (DDM) made from an extracted human tooth were simultaneously placed under the skin of 20 nude rats to observe the bone-forming capability [1]. In this case series, bone regeneration was evaluated using histomorphometric analysis. The latter showed an encouraging number of osteoprogenitor cells compared with the dentin particles present. Upon histological examination at 4 weeks, the deposition of new bone matrix and absorption of dentin particles were also observed. Subcutaneous implantation of PDRN and DDM resulted in excellent osteoinductive activity, inducing the growth and proliferation of fibroblasts, osteoblasts, and new bone [1].

A recent study examined the bone inductive potential of a block graft composed of hydroxyapatite/tricalcium phosphate (HA/TCP), which was treated with recombinant human bone morphogenic protein 2 (rhBMP2) or PDRN. This graft was placed in surgical holes created in the neurocranium of white rabbits [20]. In the control group, the defect was filled with the HA/TCP scaffold. In the other groups, PDRN or rhBMP2 were incorporated into the scaffold at various concentrations: 0.1 mg/mL, 1 mg/mL, 5 mg/mL, and 10 mg/mL for PDRN, and 0.01 mg/mL, 0.05 mg/mL, and 0.1 mg/mL for rhBMP2. After 8 weeks, the groups treated with 5 mg/mL and 10 mg/mL PDRN, and 0.05 mg/mL and 0.1 mg/mL rhBMP-2 exhibited significantly higher levels of new bone formation compared to the control group [20]. In conclusion, HA/TCP blocks demonstrated suitable compressive strength for clinical application and exhibited significant potential for bone regeneration when combined with the appropriate concentration of PDRN or rhBMP2 [20].

The latest animal study published to date shifted the interest toward the evaluation of surgical techniques typically performed on humans. In this study, four dogs underwent lateral sinus floor elevation simultaneously with implant insertion. PDRN in addition to a synthetic bone substitute were used as grafting materials to investigate early bone formation [19]. The primary objective was to evaluate the osteoinductive effect of PDRNs below the Schneiderian membrane and, consequently, to assess how PDRNs could promote bone neoformation in areas with low osteogenic potential, as the maxillary sinus [19]. Histomorphological analyses revealed that in the test group treated with PDRN, there was a statistically significant increase in bone regeneration at the apical level compared to the control group, which was treated solely with synthetic collagenated bone. Additionally, within the test group, the apical region of the augmented area showed a stronger inclination towards osteogenesis in comparison to the coronal region [19].

From the analysis of the available in vitro literature, it can be concluded that PDRN may represent a novel material that could significantly stimulate osteoblastic proliferation within the first 6 days by reducing the G phase of mitosis. In addition, the use of PDRN in combination with biological and biocompatible scaffolds based on PLGA, mMH, bECM, and BMP-2 seems to improve regenerative capabilities by reducing gene expression related to inflammatory processes, osteoclastogenesis, as well as stimulating angiogenesis, which is essential for wound healing and bone repair, and osteogenesis. These findings have been corroborated in different animal species such as rats, rabbits, and dogs, following the creation of critical size defects or maxillary sinus floor elevation. Histological analyses confirmed how the application of PDRN with biocompatible scaffolds or collagenated synthetic bone allows to significantly increase the amount and density of newly formed bone and the anti-inflammatory and neo-angiogenic effects. The effects of PDRN on bone regeneration should be explored in future human randomized controlled clinical trials to confirm the promising results reported herein.

Table 3 shows an overview of the main results obtained with this scoping review.

This review has some limitations. The manufacturers, concentrations, carriers, and doses employed in the studies are heterogeneous. This could have affected the results obtained. Therefore, the minimum effective concentration of PDRN should be investigated to determine a concentration/effect relationship. Another limitation was that bone healing times were hardly comparable, as animal models have different bone metabolisms depending on the animal chosen.

Future human studies are thus needed to determine the proper clinical application of PDRNs in oral bone regeneration and to confirm the results obtained from the in vitro and animal studies analyzed in this systematic review.

## 5. Conclusions

This scoping review found that in the analyzed in vitro and in vivo studies:

1. enzymatic degradation of PDRN generates biologically active metabolites that interact with various receptors including purinergic adenosine A2A receptors.
2. Activation of these receptors promotes angiogenesis, osteoblast migration, proper extracellular matrix deposition, and reduces inflammation.
3. PDRN demonstrated a high therapeutic effect, low immunogenicity, and absence of side effects, regardless of the route of administration.
4. PDRN application with biocompatible scaffolds or collagenous synthetic bone allows the amount and density of newly formed bone to be significantly increased.

## Figures and Tables

**Table 1:** Data collection and outcomes of in vitro studies.

Article	Cell Model	PDRN Employed	Experimental Group	Follow-Up	Sample Analysis	Results
Guizzardi et al., 2003 [15]	Human osteoblasts obtained from jawbone specimens subsequently surgical intervention on a 5-year-old patient. The gender of the donor is not specified in the article.	PDRN at the concentration of 100 μg/mL distributed by Mastelli, Sanremo, Italy	PDRN stimulation on osteoblasts - FBS 1% (CTR) - FBS 5% (CTR) - FBS 10% (CTR) - PDRN + FBS 1%, - PDRN + FBS 5% - PDRN + FBS 10%	PDRN stimulation on osteoblasts 0, 2, 4, 6 days	PDRN stimulation on osteoblast - Cell Culturing: Osteoblasts cultured in DMEM medium with ascorbic acid. PDRN’s impact on cell growth measured by direct counting. Cell growth assessed in DMEM with varying FBS levels (1%, 5%, 10%) and ascorbic acid. - Test sample: Cells treated with PDRN (100 μg/mL) in DMEM medium with various FBS concentrations (1%, 5%, 10%) and 250 μg ascorbic acid for 2, 4, and 6 days. - Control Sample: Cells not treated with PDRN.	PDRN stimulation on osteoblast - PDRN has no effect in cultures without FBS. - PDRN at a high FBS concentration (10%) significantly increases osteoblast growth, peaking at 6 days with a 21% increase.
			DMPX Inhibitors effect - PDRN (CTR) - PDRN + DMPX 50 μM	DMPX Inhibitors effect 0, 2, 4, 6 days	DMPX Inhibitors effect Growth rate during PDRN treatment was assessed with and without DMPX (an A2 receptor inhibitor) - In the test group, cells were treated with PDRN + DMPX for 2, 4, and 6 days - the control group received only PDRN for the same duration.	DMPX Inhibitors effect On day 6, DMPX-treated cells presented a reduction of 42.9% in comparison to the control samples.
			Suramin inhibitors effect - PDRN (CTR) - PDRN + Suramin 10 μM - PDRN + Suramin 50 μM - PDRN + Suramin 100 μM	Suramin inhibitors effect 0, 2, 4, 6 days	Suramin inhibitors effect Growth inhibition during PDRN treatment with Suramin (a specific P2 inhibitor) was assessed. - In the test group, cells were treated with PDRN + Suramin at various concentrations (10/50/100 μM) for 2, 4, and 6 days. - The control group received only PDRN for the same duration.	Suramin inhibitors effect The addition of this inhibitor at 10 μM suramin had no significant effect on PDRN induced cell growth. Higher suramine concentrations determined a strong reduction in cell number also in comparison to control just after 48 h.
			ALP - CTR PDRN	ALP 0, 2, 4, 6, 8, 10 days	ALP An increase in alkaline phosphatase activity indicates an increase in cell duplication. - In test group cells were treated for 2, 4, 6, 8 and 10 days with PDRN 100 μg/mL in DMEM complete medium plus 10% FBS and ascorbic acid 250 μg. - In control sample, cells were not treated with PDRN, cells growth was evaluated in DMEM plus FBS 10% and ascorbic acid 250 μg.	ALP The PDRN-treated cells examined on day 6 present a significantly lower phosphatase activity when compared with controls (*p* < 0.01). While there was not significant discrepancy between the two groups on day 10.
Kim et al., 2021 [17]	hBMSCs The article does not specify how these cells were obtained.	PDRN distributed by Goldbio St. Louis, MO, USA. The concentration of PDRN used was not reported.	- PLGA (CTR) - PME: PLGA (P) scaffold was combined with mMH (M) and bECM € - PMED: PLGA (P) scaffold was combined with MH (M), bECM € and the bioactive PDRN (P)		Scaffold Characterization WCA was conducted to evaluate the wettability of the scaffold.	Scaffold Characterization The PMEP scaffold has more hydrophilic property than the PLGA and PME ones.
				Biocompatibility of the scaffold 1, 3, and 7 days	Biocompatibility of the scaffold The biocompatibility of the scaffolds was evaluated since LIVE/DEAD staining [calceinAM (acetoxymethyl ester) and ethidium homodimer 1 (EthD-1)].	Biocompatibility of the scaffold The population of live cells was getting increased in the PME and the PMEP than the PLGA at 1, 3, and 7 days, respectively. The cell viability on the PME and particularly, the PMEP scaffold was remarkably enhanced for 7 days, *p* < 0.05 and *p* < 0.001, respectively.
				Angiogenesis and ant-inflammation properties 7 and 21 days	Angiogenesis and ant-inflammation properties RNA Extraction and Quantitative Real-Time PCR (qRT-PCR) were conducted to determine the expression of inflammation and angiogenesis-related genes on 3D scaffolds with hBMSCs.	Angiogenesis and ant-inflammation properties - The PME scaffold restricted the expression of inflammatory genes, IL-6 and IL-1*β*, compared to the PLGA scaffold. - The PMEP scaffold statistically significantly suppressed the abovementioned gene compared with PME. - The PME scaffold exhibited a negligible difference in the expression of angiogenesis-related gene. - The PMEP scaffold promoted the highest angiogenesis-related gene (VEGF and MMP2) expression on both days. The expression of these genes was statistically significant compared with the other groups.
				Confirm of angiogenic ability and wound healing 7 and 21 days	Confirm of angiogenic ability and wound healing The angiogenesis ability was examined by tubule-forming assay with HUVECs. This samples were stained with calcein AM then photographed with a fluorescence microscope.	Confirm of angiogenic ability and wound healing When PDRN-treated, HUVECs had formed a significant number of branch points and longer lengths of tubes. On the same side, because PDRN could enhance the growth and migratory ability of hBMSCs, the wound closure rates also highly increased to 34.8 and 31.9% in PDRN-treated groups compared to control at 24 and 48 h, respectively.
				Osteogenesis in 3D Scaffold 7 and 21 days	Osteogenesis The osteogenic capacity of the scaffold was assessed through qRT-PCR on osteogenesis-related genes expression, such as RUNX2, OPN, osteocalcin OCN using hfbMSCs.	Osteogenesis in 3D Scaffold The PME scaffold could induce osteogenic differentiation of hBMSCs effectively, and by adding PDRN (PMEP), the osteogenesis was more enhanced. The results exhibited that the PMEP scaffold significantly up-regulated RUNX2, OPN and OCN at all days.
				Attenuation of Osteoclastogenesis Not reported	Attenuation of Osteoclastogenesis The attenuation of ostotoclastogenesis by PDRN was evaluated using macrophages cells, in detail RAW264.7. RAW264.7 were induced to differentiate into osteoclasts by stimulation of receptor activator of RANKL and M-CSF. These macrophages were investigated using TRAP staining and activity assay.	Attenuation of Osteoclastogenesis Bioactive molecules secreted by scaffold PME and PMEP statistically significantly attenuated differentiation into osteoclasts of RAW264.7 cells by 31.7 and 74.4%, respectively, compared with control.
Kim et al., 2021 [18]	hBMSCs The article does not specify how these cells were obtained.	1 mg of PDRN dissolved in 1 mL of nuclease-free water. The PDRN was distributed by Goldbio St. Louis, MO, USA.	- PLGA (CTR) - PME + BMP2 - PME + PDRNPME + NC: the NC was formed by the union of PDRN and BMP2	Not reported	Angiogenic efficacy of NC Immunocytochemistry with an anti VEGF antibody using HUVECs was conducted to evaluate increased VEGF production due to PDRN. To confirm the angiogenic effect on NC, was executed qRT-PCR on angiogenesis.	Angiogenic efficacy of NC The PDRN- and NC-treated groups demonstrated an increase in the number of VEGF positive cell observation compared to the control and BMP2-treated groups in 3 days. The gene expression levels of VEGF in the PDRN- and NC-treated groups significantly increased (*p* < 0.01 and *p* < 0.001). The group treated with NC exhibited a statistically significant increase in the expression levels of ANG2, which is one of the most prevalent angiogenic factors.
					Biocompatibility of the hybrid scaffold The biocompatibility of the hybrid scaffold with NC (PME/NC) was evaluated based on LIVE/DEAD staining [calcein AM (acetoxymethyl ester) and ethidium homodimer 1 (EthD-1)].	Biocompatibility of the hybrid scaffold with NC This analysis showed that hfMSCs were viable one day after seeding and the cells proliferated well on each scaffold, especially PME/NC.
					Osteogenic potential of the hybrid scaffold with NC The osteogenic capacity of the scaffold was assessed using qRT-PCR on osteogenesis-related genes expression, such as RUNX2, OPN, OCN and ON using hfMSCs. ALP is a marker of osteogenesis, so ALP staining was conducted on each scaffold.	Osteogenic potential of the hybrid scaffold with NC The BMP2-treated group showed a significant increase in ALP activity and mineralization. However, PDRN also affected osteogenesis compared to control. Consequently, the NC has a brilliant osteogenic ability, which is made by a combinational effect from BMP2 and PDRN. RUNX2, OPN, OCN, and ON were expressed to higher levels in the PME/NC scaffold compared to any other scaffolds.
					Attenuation of osteoclastogenesis and inflammatory gene expression The attenuation of osteoclastogenesis by PDRN and BMP-2 was evaluated using RAW264.7. RAW264.7 were induced to differentiate into osteoclasts by stimulation of RANKL and M-CSF. These macrophages were investigated using TRAP staining and activity assay. To confirm the anti-inflammatory effect on NC, was executed qRT-PCR on inflammatory gene expression.	Attenuation of osteoclastogenesis and inflammatory gene expression In the groups containing PDRN, the differentiation of osteoclast was statistically inhibited as compared with the control and BMP2 and the PDRN and NC groups decreased the expressions of IL-1*β* and IL-6.

Abbreviation: hBMSCs, human bone-marrow mesenchymal stem cells; HUVECs, human umbilical vein endothelial cells; IL-1*β*, interleukin-1*β*; IL-6, interleukin-6; M-CSF, macrophage colony-stimulating factor; mMH, magnesium hydroxide; MMP2, matrix metalloproteinase-2; NC, nanocomplex; OCN, osteocalcin; ON, osteonectin; PDRN, polydeoxyribonucleotide; PLGA, Poly(lactic-co-glycolic) acid; PME, PLGA, mMH, bECM complex; PMEP, PLGA, mMH, bECM, PDRN complex; qRT-PCR, polymerasechain reaction; RANKL, receptor activator of the nuclear factor B ligand; RUNX2, runt-related transcription factor 2; TRAP, tartrate-resistant acid phosphatase; VEF, Vascular Endothelial Growth Factor; WCA, Water Contact Angle; BMP2, bone morphogenetic protein.

**Table 2:** Data collection and outcomes of in vivo studies.

Article	Animal Model	Study Design	Surgical Procedure	PDRN	Experimental Grup	Follow-Up	Complication	Analysis of Newly Formed Bone Volume	Qualitative Histological Analysis
Kim et al., 2016 [1]	Mice (20) The gender of these animals has not been reported.	Case Series	The dorsal portion was incised, and a subcutaneous pouch was formed in both sides. DDM and PDRN was implanted into the subcutaneous pouch.	PDRN 1.875 *w*/*v*% solution distributed by Mastelli, Sanremo, Italy.	Group 1: DDM + PDRN	The animals were sacrificed at 1, 2, and 4 weeks.	Not reported.	The valuation of bone regeneration was a histomorphometric analysis. - Quantitative level of bone-forming cells around DDM The average values of this parameter were 10, 20, and 23 at 1, 2, and 4 weeks. - Area of newly formed mineralized bone to obtained image area (NB%) NB% was 7, 20, and 17% at 1, 2, and 4 weeks, respectively.	- At 1 week, a fibrous capsule which was well bounded with neighboring tissue was observed. - At 2 weeks, much greater bone-forming cells were observe than in the first week and development of the blood vessels and newly formed collagen matrix were. - At 4 weeks, the deposition and calcification of new bone matrix were observed.
Guizzardi et al., 2007 [16]	Male Sprague-Dawley Rats (32)	RCT	Two round holes in the cortical bone of both tibiae of each rat were created.	PDRN at the concentration of 95% distributed by Mastelli, Sanremo, Italy	- Group 1: CRT (8) the defects were left empty. - Group 2: HDB (8) - Group 3: PDRN (8) - Group 4: HDB + PDRN (8)	For each group 2 animals were sacrificed at 1, 2, 4, 12 weeks	No inflammatory or adverse reactions to PDRN gel and/or HDB/PDRN paste were detected; only a weak lymphocyte infiltrate was detectable at 1 week.	Not reported.	- Group 1: At 12 weeks, the bone defect had been almost completely replaced by new formed trabecular bone. - Group 2: After 12 weeks, the defect was filled by new formed bone along with the embedded HDB granules. - Group 3: After 4 weeks, new-formed trabecular bone was seen departing from the border. The defect was filled by new bone after 12 weeks. - Group 4: no granules of HDB detected outside the surgical defect and a faster formation of new bone than in the other experimental conditions.
Kim et al., 2021 [18]	Rats The gender of these animals has not been reported.	RCT	On both sides of rat calvaria, a defect (diameter 5 mm and 1.5 mm thickness) was made using micro drill and trephine bur. The scaffolds were implanted within the defect.	1 mg of PDRN dissolved in 1 mL of nuclease-free water. The PDRN was distributed by Goldbio St. Louis, MO, USA.	- Group 1: CTR the defect was left empty. - Group 2: PLGA - Group 3: PME - Group 4: PME/NC	The animals were sacrificed after 8 weeks post-operative		The valuation of bone regeneration was performed on micro-CT. Bone volume density (BV/TV%) and BMD (%) were analyzed. - In the group implanted with PME/NC, regenerated bone was detected in the defect area. In the other groups, the formation of new bone was minor. - The bone volume density for the PME/NC implanted group was significantly higher than the control (*p* < 0.0001). - The BMD of the PME/NC increased by approximately four times higher than control. In addition, the vessel volume density and vessel number were quantified at micro CT. - PME/NC appeared with numerous newly thick vessels and showed negligible difference with the control group (*p* > 0.05).	- Considerably newly developed bone tissue was observed in the defected area for the PME/NC scaffold than control. Additional immunohistochemistry analysis to assess vascularization and anti-inflammatory effect of scaffolds in vivo was performed. - In the PME/NC scaffold, the expression of angiogenetic and osteogenetic genes was statistically significantly higher than the other groups. - Also, in PME/NC groups decreased the expressions of IL-1*β* and IL-6.
Lee et al., 2022 [19]	Male Beagle Dogs (4)	RCT	Both premolars (P2, P3, and P4) in the maxilla were extracted. The alveolar ridges were allowed to heal for 2 months. At the site of extracted premolars in the maxilla, implants were placed in each dog with a sinus elevation procedure (lateral approach).	The concentration of PDRN used was not reported in this study, nor was the company that manufactured it.	- Group 1: Collagenated synthetic bone - Group 2: Collagenated synthetic bone + PDRN	The animals were sacrificed after 2 months post-operative.	None of the animals showed any serious complications, including infection and postoperative bleeding around the surgical wound area.	In this study the valuation of bone regeneration was a histomorphometric analysis. To evaluate the new bone, three rectangular area of interest (1 × 1 mm) were set within the augmented sinus area: AOI_C, AOI_M, AOI_A. - The variables AH, PH, BICp, BICa total, BICa coronal, and BICa middle did not demonstrate significant statistical differences between the control and test groups. BICa apical of samples in the test group (76.7 ± 9.3%) showed a statistically significantly higher value than that of samples in the control group (55.6 ± 22.1%; *p* = 0.038). - pNB, pRBP, and pFVT in AOI_A showed statistically significant differences between samples in the 2 groups (*p* = 0.038, *p* = 0.028, and *p* = 0.007, respectively).	In augmented area, new bone formation was observed in the augmented sinus cavity in both groups. In the test group, the apical region of the augmented area exhibited a greater tendency towards osteogenesis compared to the coronal region.
Lim et al., 2021 [20]	Rabbit (32) The gender of these animals has not been reported.	RCT	Four round-shaped borders of 7 mm diameter were designed and drawn on the calvaria bone. Additional 9 holes of 1 mm diameter were formed within each round border for enhancing bone regeneration capacity and blood supply. Prefabricated polycarbonate tubes (7 mm diameter × 5 mm height) were fitted into the 7 mm round borders, and the block-type ceramic scaffolds were designed and inserted into the tubes.	PDRN at different concentrations (0.1 mg/mL, 1 mg/mL, 5 mg/mL, and 10 mg/mL). The producing company was not reported.	- Group 1: Only Scaffold (HA/TCP) - Group 2: HA/TCP + 0.1 mg/mL PDRN - Group 3: HA/TCP + 1 mg/mL PDRN - Group 4: HA/TCP + 5 mg/mL PDRN - Group 5: HA/TCP + 10 mg/mL PDRN - Group A: HA/TCP + 0.01 mg/mL BMP2 - Group B: HA/TCP + 0.05 mg/mL BMP2 - Group C: HA/TCP + 0.1 mg/mL BMP2	8 animals were sacrificed on the 4th and 8th week post-operative.	There were no apparent abnormal symptoms of infection or inflammation on the operated sites.	The valuation of bone regeneration was a histomorphometric analysis. Percent bone volume (%) = New bone volume/Total volume in scaffold × 100 At 8 weeks, new bone formation in the groups administered with 5 mg/mL and 10 mg/mL PDRN was significantly more than that in the control group. Additionally, there was no significant difference in bone formation in the group treated with 5 mg/mL PDRN compared to that in the group treated with 10 mg/mL PDRN (*p* > 0.05). At 8 weeks post-operation, new bone formation was significantly higher in the groups administered with 0.05 and 0.1 mg/mL rhBMP-2 compared to that in the control group. The extent of bone formation differed significantly in the groups administered with 0.05 and 0.1 mg/mL of rhBMP-2, and the extent of new bone formation increased at higher concentrations (*p* < 0.05).	
Farley JR et al., 2014 [21]	Beagle Dogs (6) The gender of these animals has not been reported.	RCT	2nd and 3rd premolars in both sides of the mandible of beagle dogs were extracted. An implant was placed in each socket, in the buccal area a dehiscence defect was formed (5 mm in length and 5 mm in diameter) and a bone graft was performed.	PDRN distributed by Mastelli, Sanremo, Italy. The concentration of PDRN used was not reported.	- Group 1: Xenogenic bone graft was positioned in in the 2nd premolar region in the left of maxilla. - Group 2: Xenogenic bone graft was positioned in in the 3rd premolar region in the right of maxilla. - Group 3: Xenogenic bone graft and PDRN was positioned in the 3rd premolar region in the left maxilla. - Group 4: Xenogenic bone graft and PDRN was positioned in the 2nd premolar in the right of maxilla	2 animals were sacrificed at a time after 2, 4, 8 weeks.	One dog of the group of the 4th week showed edema in the left area of surgery.	The valuation of bone regeneration was performed on micro-CT. Bone volume ratio = Bone volume/total volume The total volume is defined as the ROI which is the inside (width: 0.4 mm/length: 3.3 mm) of the implant threads. In group 2, 4, the bone volume ratio was highest in the 8th week compared to other groups and group 2 and 4 showed 55.9% and 55.4%, respectively. But there was no significant difference among group. The number of specimens was small so statistical analysis was hard to performed.	The amount of bone formation was more in group 1, 2 than group 3, 4 at 2 weeks. At 4 weeks there was a small quantity of immature bone formation around the grafted bone.

Abbreviation: AH, augmented height; AOI_A, apical region; AOI_C, most coronal region; AOI_M, middle region; BICa%, bone-to-implant contact in augmented bone; BICp%, bone-to-implant contact in pristine bone; BMD, bone mineral density; BMP2, bone morphogenetic protein; BV, bone volume; CT, computed tomographic; DDM, demineralized dentin matrix; HA, hydroxyapatite; HDB, high temperature-deproteinated bone; IL-1*β*, interleukin-1*β*; IL-6, interleukin-6; PDRN, polynucleotides; pFVT%, Fibrovascular connective tissue area percentage; PH, protruding height; pNB%, new bone area percentage; pRBP%, residual bone graft particle area percentage; ROI, region of interest; TCP, tricalcium phosphate scaffolds; TV, tissue volume.

**Table 3:** Overview of key findings achieved through this scoping review.

Property/Characteristics	Description
Interaction with adenosine A2 receptors	PDRN appears to interact with adenosine A2 receptors and, contributing to its osteoblastic growth-stimulating effects, is supported by experimental evidence
Promotes tissue regeneration	PDRN: - stimulates osteoblastic proliferation. - contributing to osteogenesis and exhibiting osteoinductive properties by showing increased expression of osteogenesis-related genes such as RUNX2, OPN, and OCN. - promotes angiogenesis: highest angiogenesis-related gene (VEGF and MMP2) expression. - anti-inflammatory properties: increased expression of inflammatory genes, IL-6 and IL-1*β*. - attenuated differentiation into osteoclasts.

## Data Availability

Upon request to the corresponding author, the data are available for use.

## Supplementary Materials

The following supporting information can be downloaded at: https://www.mdpi.com/article/10.3390/dj11120280/s1, Table S1. PRISMA-ScR Checklist.

## Abbreviations

AH	augmented height
ALP	alkaline phosphatase
ANG2	angiopoietin-2
AOI_A	apical region
AOI_C	most coronal region
AOI_M	middle region
bECM	bone-extracellular matrix
BICa%	bone-to-implant contact in augmented bone
BICp%	bone-to-implant contact in pristine bone
BMD	bone mineral density
BMP2	bone morphogenetic protein
BV	bone volume
CT	computed tomographic
DDM	demineralized dentin matrix
FBS	foetal bovine serum
HA	hydroxyapatite
hBMSCs	human bone-marrow mesenchymal stem cells
HDB	high temperature-deproteinated bone
HUVECs	humanumbilical vein endothelial cells
IL-1*β*	interleukin-1*β*
IL-6	interleukin-6
M-CSF	macrophage colony-stimulating factor
mMH	magnesium hydroxide
MMP2	matrix metalloproteinase-2
NC	nanocomplex
OCN	osteocalcin
ON	osteonectin
PDRN	polydeoxyribonucleotide
pFVT%	fibrovascular connective tissue area percentage
PH	protruding height
PLGA	poly(lactic-co-glycolic) acid
PME	PLGA, mMH, bECM complex
PMEP	PLGA, mMH, bECM, PDRN complex
pNB%	new bone area percentage
pRBP%	residual bone graft particle area percentage
qRT-PCR	polymerasechain reaction
RANKL	receptor activator of the nuclear factor B ligand
ROI	region of interest
RUNX2	runt-related transcription factor 2
TCP	tricalcium phosphate scaffolds
TRAP	tartrate-resistant acid phosphatase
TV	tissue volume
VEF	vascular endothelial growth factor
WCA	water contact angle

## References

[1] Kim S.-K., Huh C.-K., Lee J.-H., Kim K.-W., Kim M.-Y. (2016). Histologic study of bone-forming capacity on polydeoxyribonucleotide combined with demineralized dentin matrix. Maxillofac. Plast. Reconstr. Surg..

[2] Lee D.-W., Hyun H., Lee S., Kim S.Y., Kim G.-T., Um S., Hong S.O., Chun H.J., Yang D.H. (2019). The effect of polydeoxyribonucleotide extracted from salmon sperm on the restoration of bisphosphonate-related osteonecrosis of the jaw. Mar. Drugs.

[3] Squadrito F., Bitto A., Irrera N., Pizzino G., Pallio G., Minutoli L., Altavilla D. (2017). Pharmacological Activity and Clinical Use of PDRN. Front. Pharmacol..

[4] Colangelo M.T., Galli C., Guizzardi S. (2020). The effects of polydeoxyribonucleotide on wound healing and tissue regeneration: A systematic review of the literature. Regen. Med..

[5] Ko I.-G., Jin J.-J., Hwang L., Kim S.-H., Kim C.-J., Jeon J.W., Chung J.-Y., Han J.H. (2021). Adenosine A2A receptor agonist polydeoxyribonucleotide ameliorates short-term memory impairment by suppressing cerebral ischemia-induced inflammation via MAPK pathway. PLoS ONE.

[6] Galeano M., Bitto A., Altavilla D., Minutoli L., Polito F., Calò M., Cascio P.L., D’Alcontres F.S., Squadrito F. (2008). Polydeoxyribonucleotide stimulates angiogenesis and wound healing in the genetically diabetic mouse. Wound Repair Regen..

[7] Veronesi F., Dallari D., Sabbioni G., Carubbi C., Martini L., Fini M. (2017). Polydeoxyribonucleotides (PDRNs) from Skin to Musculoskeletal Tissue Regeneration via Adenosine A(2A) Receptor Involvement. J. Cell. Physiol..

[8] Buffoli B., Favero G., Borsani E., Boninsegna R., Sancassani G., Labanca M., Rezzani R., Nocini P.F., Albanese M., Rodella L.F. (2017). Sodium-DNA for Bone Tissue Regeneration: An Experimental Study in Rat Calvaria. BioMed Res. Int..

[9] Koo Y., Yun Y. (2016). Effects of polydeoxyribonucleotides (PDRN) on wound healing: Electric cell-substrate impedance sensing (ECIS). Mater. Sci. Eng. C.

[10] Tricco A.C., Lillie E., Zarin W., O’Brien K.K., Colquhoun H., Levac D., Moher D., Peters M.D.J., Horsley T., Weeks L. (2018). PRISMA Extension for Scoping Reviews (PRISMA-ScR): Checklist and Explanation. Ann. Intern. Med..

[11] Bateson M. (2004). Systematic Reviews to Support Evidence-Based Medicine: How to Review and Apply Findings of Healthcare Research. Postgrad. Med. J..

[12] Al-Ardah A.J., AlHelal A., Proussaefs P., AlBader B., Al Humaidan A.A., Lozada J. (2017). Managing Titanium Mesh Exposure with Partial Removal of the Exposed Site: A Case Series Study. J. Oral Implant..

[13] Munn Z., Stern C., Aromataris E., Lockwood C., Jordan Z. (2018). What kind of systematic review should I conduct? A proposed typology and guidance for systematic reviewers in the medical and health sciences. BMC Med. Res. Methodol..

[14] Jung J., Lim H.S., Lee D.-W. (2018). Polydeoxyribonucleotide, as a novel approach for the management of medication-related osteonecrosis of the jaw: A preliminary observational study. J. Korean Dent. Sci..

[15] Guizzardi S., Galli C., Govoni P., Boratto R., Cattarini G., Martini D., Belletti S., Scandroglio R. (2003). Polydeoxyribonucleotide (PDRN) promotes human osteoblast proliferation: A new proposal for bone tissue repair. Life Sci..

[16] Guizzardi S., Martini D., Bacchelli B., Valdatta L., Thione A., Scamoni S., Uggeri J., Ruggeri A. (2007). Effects of heat deproteinate bone and polynucleotides on bone regeneration: An experimental study on rat. Micron.

[17] Kim D.-S., Lee J.-K., Jung J.-W., Baek S.-W., Kim J.H., Heo Y., Kim T.-H., Han D.K. (2021). Promotion of Bone Regeneration Using Bioinspired PLGA/MH/ECM Scaffold Combined with Bioactive PDRN. Materials.

[18] Kim D.-S., Lee J.-K., Kim J.H., Lee J., Kim D.S., An S., Park S.-B., Kim T.-H., Rim J.S., Lee S. (2021). Advanced PLGA hybrid scaffold with a bioactive PDRN/BMP2 nanocomplex for angiogenesis and bone regeneration using human fetal MSCs. Sci. Adv..

[19] Lee D., Lee J., Koo K.-T., Seol Y.-J., Lee Y.-M. (2021). The impact of polydeoxyribonucleotide on early bone formation in lateral-window sinus floor elevation with simultaneous implant placement. J. Periodontal Implant. Sci..

[20] Lim H.-K., Kwon Y.-J., Hong S.-J., Choi H.-G., Chung S.-M., Yang B.-E., Lee J.-H., Byun S.-H. (2021). Bone regeneration in ceramic scaffolds with variable concentrations of PDRN and rhBMP-2. Sci. Rep..

[21] Ji-Young L., Young-Kyun K., Pil-Young Y., Ju-Cheol P., Kyo-Jin A., Sooyeon K. (2014). Evaluation of bone healing capacity of xenogenic tooth bone graft material with polydeoxyribonucleotide in bone defect surrounding an implant. Oral Biol. Res..

[22] Baj A., Trapella G., Lauritano D., Candotto V., Mancini G.E., Giannì A.B. (2016). An overview on bone reconstruction of atrophic maxilla: Success parameters and critical issues. J. Biol. Regul. Homeost. Agents.

[23] Ra G., Wo Q. (2021). Bone regeneration in dentistry: An overview. Biol. Regul. Homeost. Agents.

[24] Polo-Corrales L., Latorre-Esteves M., Ramirez-Vick J.E. (2014). Scaffold design for bone regeneration. J. Nanosci. Nanotechnol..

[25] Gallo S., Pascadopoli M., Pellegrini M., Pulicari F., Manfredini M., Zampetti P., Spadari F., Maiorana C., Scribante A. (2022). Latest Findings of the Regenerative Materials Application in Periodontal and Peri-Implant Surgery: A Scoping Review. Bioengineering.

[26] Oguić M., Čandrlić M., Tomas M., Vidaković B., Blašković M., Radetić A.T.J., Cvek S.Z., Kuiš D., Peloza O.C. (2023). Osteogenic Potential of Autologous Dentin Graft Compared with Bovine Xenograft Mixed with Autologous Bone in the Esthetic Zone: Radiographic, Histologic and Immunohistochemical Evaluation. Int. J. Mol. Sci..

[27] A Urban I., Nagursky H., Lozada J.L. (2011). Horizontal ridge augmentation with a resorbable membrane and particulated autogenous bone with or without anorganic bovine bone-derived mineral: A prospective case series in 22 patients. Int. J. Oral Maxillofac. Implant..

[28] Orriss I.R., Knight G.E., Ranasinghe S., Burnstock G., Arnett T.R. (2006). Osteoblast responses to nucleotides increase during differentiation. Bone.

